# Retroperitoneal abscess as a presentation of colon cancer: The largest case set analysis to date, which extracted from our unit and the literature

**DOI:** 10.3389/fonc.2023.1198592

**Published:** 2023-10-24

**Authors:** Junmin Zhou, Songlin Wan, Chunguang Li, Zhao Ding, Qun Qian, Hao Yu, Daojiang Li

**Affiliations:** ^1^ Department of Colorectal and Anal Surgery, Zhongnan Hospital, Wuhan University, Wuhan, Hubei, China; ^2^ Departments of Anorectal Surgery, Xianning Central Hospital, The First Affiliated Hospital of Hubei University of Science and Technology, Xianning, Hubei, China; ^3^ Department of Colorectal and Anal Surgery, Clinical Center of Intestinal and Colorectal Diseases of Hubei Province, Wuhan, Hubei, China; ^4^ Hubei Key Laboratory of Intestinal and Colorectal Diseases, Zhongnan Hospital, Wuhan University, Wuhan, Hubei, China; ^5^ Department of Colorectal and Anal Surgery, Quality Control Center of Colorectal and Anal Surgery of Health Commission of Hubei Province, Wuhan, Hubei, China; ^6^ Department of Radiology, Zhongnan Hospital of Wuhan University, Wuhan, Hubei, China

**Keywords:** colon cancer, colon tumor, abscess, retroperitoneal abscess, misdiagnosis, psoas abscess

## Abstract

**Objective:**

Colon cancer with retroperitoneal abscess is a rare and easily misdiagnosed disease and has only been reported via case. There is an urgent need to conduct a dataset analysis for such patients, which is crucial to improving the survival rate and quality of life of these patients

**Methods:**

Patients with colon cancer associated with retroperitoneal abscess were extracted from our hospital and the PubMed, EMBASE and Web of Science databases. Clinical information, including the patients’ basic characteristics, clinical symptoms, laboratory tests, imaging examinations, treatment methods and prognosis was analyzed.

**Results:**

Sixty-one patients were analyzed, with an average age of 65 years. The proportions of right and left colon cancers were 63.9% and 36.1%, respectively. A total of 98.0% of the patients had adenocarcinoma. Many patients have insidious symptoms such as fever and weight loss. At the first medical visit, pain was the most common symptom (71%), with pain in the thigh (21.8%), abdomen (21.8%), and waist and back (14.5%) ranking among the top three. The misdiagnosis rate of the patients referred to our department was 75%, while the overall misdiagnosis rate in the literature was 43.9%. Laboratory tests show that these patients often have elevated white blood cells and anemia. CT examination showed that 87.2% of patients had an iliopsoas muscle abscess, and tumors were not simultaneously detected in 37.2%. A total of 33.9% of patients had local abscesses of the iliopsoas muscle, 26.4% had drainage into the subcutaneous tissue of the waist and upper buttocks, and 22.6% had drainage around the adductor muscle group of the thigh. These patients have a variety of treatments, and many patients have undergone multiple and unnecessary treatments. Thirteen patients died after surgery, and 6 died in the hospital, of whom four were patients undergoing direct surgery, and the other 7 died after discharge due to cachexia.

**Conclusion:**

Colorectal cancer with retroperitoneal abscess is a relatively rare and easily misdiagnosed subtype of colon cancer. It is more likely to occur in right-sided colon adenocarcinoma. The main clinical symptom is pain caused by the drainage of pus to the corresponding areas of the waist, abdomen, and legs. CT is the preferred diagnostic method. Actively treating the abscess and then transitioning to standard colon cancer treatment can prevent patient death and improve treatment quality.

## 1 Introduction

Colon cancer is a common malignant tumor; in recent years, many evidence-based medical treatment methods from multiple disciplines have significantly improved the survival rate and quality of life of colon cancer patients; and the medical and surgical treatment of colon cancer has become increasingly standardized (1). However, due to individual differences and other reasons, some patients often have complications such as perforation, bleeding, obstruction and abscess formation, which leads to these patients not being able to immediately undergo standardized treatment in accordance with the guidelines (2). Therefore, how to deal with these complications in a timely and effective manner and get the patient’s tumor treatment to return to the standardized treatment has an important impact on the prognosis of patients. In this article, we present an analysis of a very rare complication of colon cancer, retroperitoneal abscess.

The colon is an interperitoneal organ, and the ascending and descending colons are located behind the peritoneum (3). Tumors in these two places will cause retroperitoneal abscesses due to tumor invasion and perforation. We found that retroperitoneal abscess due to colon cancer has only been reported via case reports and is a rare condition with an incidence of 0.3%, according to some literature reports (4). Through these reports, it is clear that due to the lack of treatment experience available for reference, there are enormous differences in the treatment methods and treatment outcomes of such patients, and many of these cases are misdiagnosed. Therefore, there are many challenges for clinicians regarding how to deal with such patients accurately, effectively and in a timely manner. Based on this, we retrospectively compared and analyzed the basic characteristics, clinical symptoms, laboratory tests, imaging examinations, treatment methods and prognosis of 4 patients in our hospital and 57 patients reported in the literature. Through these comparative analyses, this paper attempts to provide an optimal reference for the diagnosis and treatment of retroperitoneal abscess associated with colon cancer.

## 2 Materials and methods

We extracted 1013 colon cancer patients admitted to our hospital from October 2017 to October 2022, and the first two doctors searched for colon cancer cases associated with abscess through CT and MRI imaging information systems. The search range included descriptive texts of the imaging results and diagnostic conclusions. Next, the patients who were initially screened were manually queried through the clinical information system to confirm whether the patient had a retroperitoneal abscess, and for the final confirmed patients, detailed clinical information, including the basic information of the patient, tumor location, abscess location, scope, symptoms, physical examination, microorganisms, imaging tests, treatment methods, etc., were recored. Follow-up of the patients was done by one doctor. Literature reading and information extraction were completed by 3 doctors. The scope of of search included PubMed, EMBASE and Web of Science, updated on February 10, 2023, and was first completed by 3 doctors alone. The extracted information included basic information on the patients, tumor location, abscess location, scope, symptoms, physical examination, microorganisms, imaging tests, treatment methods. Finally, all authors complemented and verified the collected data. In order to effectively demonstrate the characteristics of patients with retroperitoneal abscess caused by colon cancer, we conducted a comprehensive analysis in five major aspects, including patient characteristics, clinical symptoms, laboratory examinations, imaging studies, and treatment. At the beginning of each aspect, we first analyzed the characteristics of patients from our institution, and then provided detailed descriptions based on the extracted cases from the literature.

## 3 Result

### 3.1 Patient characteristics

A total of 1013 patients with colon cancer were extracted from the CT and MRI information system of our hospital, and 4 cases were confirmed to be colon cancer associated with a retroperitoneal abscess, with an incidence rate of 0.39%. The detailed characteristics of the 4 patients are listed in **Table 1**. We extracted 51 case reports of retroperitoneal abscesses caused by colon cancer from the literature, involving 57 patients, whose detailed features are listed in **Table 2**. Of these patients, age was provided in 60 patients, the age range of these patients was 27~86 years old, the average age was 65 years, and the cohort included 27 women (45%) and 33 men (55%). The most reported country was Japan with 15 cases (24.6%), followed by China with 13 cases (21.3%), the United States with 11 cases (18.0%), the United Kingdom with 6 cases (9.8%), India with 4 cases (6.6%), Italy with 3 cases (4.9%), Greece and South Korea with 2 cases each (3.3%), and Croatia, North Macedonia, Spain, Tunis and Brazil with 1 case each. There were 39 cases (63.9%) in which the tumor was located in the right colon, including 20 cases (32.8%) in the ileocecal region, 14 cases in the ascending colon (23.0%), 5 cases in the appendix (8.2%), and 22 cases (36.1%) in the left colon, including 9 cases (14.8%) in the sigmoid colon and 13 cases (21.3%) in the descending colon. Of the 4 patients from our department, two had a history of previous hypertension and diabetes, 1 had a family history, and most of the 57 case reports did not provide a history or family history. In fifty-one patients, the information regarding the histologic type of tumor was provided, 50 of these patients had adenocarcinoma (98.0%) and one had a neuroendocrine tumor (2%).

**Table 1:** Patient characteristics of our department.

No.	age	sex	country	Tumor Site	Histologic Type	previous history	family history
1	45	Male	China	descending colon	Adenocarcinoma	no	yes
2	54	Male	China	descending colon	Adenocarcinoma	no	no
3	67	Male	China	ascending colon	Mucinous adenocarcinoma	Hypertension and diabetes	no
4	55	Female	China	descending colon and sigmoid	Adenocarcinoma	Hypertension and diabetes	no

**Table 2:** Patient characteristics from case report.

No.	Reference	age	sex	country	Tumor Site	Histologic Type	previous history	family history
	H. Kobayashi2001 (4)	72	female	Japan	Cecum	well-differentiated adenocarcinoma(IIIa)	unclear	unclear
2	Peterson CM 1983 (5)	62	male	USA	ascending colon	adenocarcinoma	unclear	unclear
3	J.LLAUGER,1987 (6)	55	male	Spain	ascending colon.	poorly differentiated adenocarcinoma	unclear	unclear
4	**Dean D.T. Maglinte 1983 (** 7)	76	male	USA	descending colon and sigmoid	/	unclear	unclear
5	**Dean D.T. Maglinte 1983 (** 7)	64	male	USA	hepatic flexure	/	unclear	unclear
6	**Dean D.T. Maglinte1983** (7)	65	male	USA	appendix	/	unclear	unclear
7	Lopa patel 2018 (8)	69	male	UK	caecal	/	unclear	unclear
8	Nigel Yong Boon Ng 2015 (9)	55	male	UK	caecal	moderated differentiated adenocarcinoma (Dukes C1 or T3N1)	unclear	unclear
9	Rob Bisset 2010 (10)	81	female	UK	caecal/ascending	moderately differentiated mucinous colonic adenocarcinoma (Dukes B, pT3N0M1)	unclear	unclear
10	G N Mann 1997 (11)	47	male	USA	caecal	Adenocarcinoma(Dukes B)	unclear	unclear
11	Zeljko Reiner 2015 (12)	65	female	Croatia	appendix	well differentiated adenocarcinoma	unclear	unclear
12	Kiyoshi Maeda 2022 (13)	65	female	Japan	cecum	mucinous cystadenocarcinoma	unclear	unclear
13	Kang Nyeong Lee 2008 (14)	27	female	Korean	descending colon	mucinous adenocarcinoma	unclear	unclear
14	Nandhagopal Vijayaraghavan 2014 (15)	50	female	India	cecum	adenocarcinoma	unclear	unclear
15	Jun-Young Yang 2011 (16)	44	male	korea	descending colon	adenocarcinoma	unclear	unclear
16	Xianlin Zeng 2017 (17)	47	female	China	sigmoid colon	metastatic poorly differentiated carcinoma	unclear	unclear
17	**Atsushi Okita 2007 (** 18)	77	male	Japan	cecal	moderately differentiated adenocarcinoma (T3N1M0)	unclear	unclear
18	**Atsushi Okita 2007 (** 18)	85	female	Japan	appendix	well differentiated adenocarcinoma(T3N1M0)	unclear	unclear
19	**Atsushi Okita 2007 (** 18)	50	female	Japan	sigmoid colon	moderately differentiated adenocarcinoma(T3N0M1)	unclear	unclear
20	M T Chuang 1995 (19)	64	male	China	appendix	mucinous cystadenocarcinoma of appendix	unclear	unclear
21	Sugato Nawa 2005 (20)	76	female	Japan	ascending colon	moderately differentiated adenocarcinom(stageII in UICC criteria)	unclear	unclear
22	Mithun S. Jakkan 2018 (21)	86	female	India	ascending colon	/	unclear	unclear
23	R C S Khandelwal 2021 (22)	52	female	India	appendix	/	unclear	unclear
24	Shotaro Harada 2017 (23)	64	male	Japan	ascending colon cancer	moderately differentiated adenocarcinoma	unclear	unclear
25	Alban Cacurri 2014 (24)	67	male	Italy	descending colon	adenocarcinoma(T4 N2 M1)	unclear	unclear
26	**Wen KM 2021 (** 25)	61	female	China	Ascending colon	mucinous adenocarcinoma.	unclear	unclear
27	**Wen KM 2021 (** 25)	75	male	China	Ascending colon	mucinous adenocarcinoma	unclear	unclear
28	**Wen KM 2021 (** 25)	73	female	China	Ascending colon	mucinous adenocarcinoma(T4bN1aM0)	unclear	unclear
29	Osama Mosalem 2020 (26)	68	male	USA	descending colon	adenocarcinoma	unclear	unclear
30	Daniel Paramythiotis 2020 (27)	75	female	Greece	ascending colon	adenocarcinoma	unclear	unclear
31	Giovanni Terrosu 2010 (28)	78	male	Italy	cecum	adenocarcinoma(T4N1Mx)	unclear	unclear
32	Vasiliki Galani 2020 (29)	61	female	Greece	ascending colon and sigmoid	poorly differentiated adenocarcinoma of the ascending colon and sigmoid, with choriocarcinomatous differentiation(T4N2M1)	unclear	unclear
33	Raja Kalayarasan 2021 (30)	70	female	India	cecum	Mixed neuroendocrine nonneuroendocrine neoplasms(T4a N0)	unclear	unclear
34	Shunki Iemura 2022 (31)	80	male	Japan	cecal	/	unclear	unclear
35	Renata Tabola 2018 (32)	67	male	Italy	descending colon	/	unclear	unclear
36	archik Das 2017 (33)	69	male	UK	caecal	adenocarcinoma (T4N1M1)	unclear	unclear
37	Harunobu Sato 2019 (34)	78	male	Japan	hematogenous metastasis to jejunum from sigmoid colon cancer	moderately differentiated adenocarcinoma	unclear	unclear
38	Yuji Takakura 2009 (35)	67	male	Japan	sigmoid colon	T4N1M1	unclear	unclear
39	Haithem Zaafouri 2015 (36)	83	female	Tunis	sigmoid colon	adenocarcinoma.	unclear	unclear
40	Hiroki Imamura 2020 (37)	66	female	Japan	descending colon	T4bN0M0	unclear	unclear
41	G R Avery 1988 (38)	71	male	UK	sigmoid	metastatic adenocarcinoma	colon cancer	unclear
42	T P Lam 1996 (39)	/		China	sigmoid	/	unclear	unclear
43	R. V. Pandini 2020 (40)	63	female	Brazil	descending colon	adenocarcinoma	hypertension	unclear
44	Yuji Haruki 2022 (41)	61	male	Japan	descending colon	well-differentiated adenocarcinoma	unclear	unclear
45	Andrej Nikolovski 2021 (42)	61	female	North Macedonia	descending colon	moderately differentiated obstructive adenocarcinoma(T4a N1c M0)	unclear	unclear
46	Yasuhiro Fujiwara 2014 (43)	65	female	Japan	cecum	well-differentiated tubular adenocarcinoma	unclear	unclear
47	Hanlei Zhou 2018 (44)	68	male	China	ileocecus	mucinous adenocarcinoma (T4bN0M0)	unclear	unclear
48	**John P. Welch 1976 (** 45)	77	male	USA	descending colon	adenocarcinoma grade II	unclear	unclear
49	**John P. Welch 1976 (** 45)	80	male	USA	cecum	adenocarcinoma grade II	unclear	unclear
50	Laura S. Heidelberg 2020 (46)	84	male	USA	proximal right colon	/	Anemia	no
51	Takayuki Yamada,2002 (47)	62	male	Japan	ascending colon	well-differentiated adenocarcinoma	unclear	unclear
52	Naoki Aomatsu 2017 (48)	46	male	Japan	cecum and ascending colon	T4bN1M0	unclear	unclear
53	Conor D Marron 2006 (49)	52	male	UK	caecum	/	unclear	unclear
54	Joslin Stanton 2022 (50)	46	female	USA	sigmoid colon	poorly differentiated adenocarcinoma of the sigmoid	unclear	no
55	Wenzhou Huang 2016 (51)	64	female	China	cecum	well differentiated adenocarcinoma	unclear	unclear
56	YU-PIN HO,2004 (52)	78	female	China	ascending colon	moderately differentiated adenocarcinoma(DUKES’ C2)	hypertension	unclear
57	Tejas Raiyani,2011 (53)	65	female	USA	ceacal	Moderately differentiated adenocarcinoma	unclear	unclear

### 3.2 Insidious and clinical symptoms before treatment

As shown in **Table 3**, two of the four patients from our department experienced physical discomfort but did not seek medical attention. The patient of case 1 had a change in stool habits for half a year, and the patient of case 4 had sour pain and discomfort in the left lower abdominal groin region for 11 days. Two patients received medical attention due to abdominal pain, one patient received medical attention due to abdominal pain and an abdominal mass, and one patient received medical attention due to an abdominal fluctuating mass. During the first medical visit, three of them (75%) had a misdiagnosis, and the patient in Case 3 was repeatedly hospitalized four times due to the misdiagnosis. During the physical examinations, all four patients were shown to have abdominal masses and local tenderness. **Stable 1** shows the clinical characteristics of such patients in the literature. Thirty patients described early insidious symptoms, but did not seek medical attention. The most common symptoms were fever (9/30, 30%) and weight loss (8/30, 23.3%), followed by pain or stool habit changes in 6 (20%) patients, anemia in 4 (13.3%) patients, and fatigue in 3 (10%) patients, and the duration of the hidden symptoms ranged from 5 days to 3 years. Fifty-five patients were described their clinical symptoms at their first visit to the hospital. Pain was the most obvious symptom (39/55, 71%), and the pain sites were as follows: thigh (12/55, 21.8%), abdomen (12/55, 21.8%), waist and back (8/55, 14.5%), groin (7/55, 12.7%), buttock (6/55, 10.9%), flank (5/55, 9%) and calf (3/55, 5.5%). Other symptoms included swelling (13/55, 23.6%), weakness and lower limb activity disorders (10/55, 18.2%), fever (9/55, 16.3%), digestive symptoms (8/55, 14.5%), erythema (6/55, 10.9%), 4 masses (4/55, 7.2%), and fistula nonunion (2/55, 3.6%). Of the 57 patients, 25 (43.9%) had a misdiagnosis at their first visit, with a frequency of 1-3 misdiagnosises. Twenty-two (38.6%) patients had a clear or highly suspected diagnosis of colon cancer, and in 10 (17.5%), it could not be inferred from the literature whether there was a misdiagnosis. Necrotizing fasciitis、local abscess and infection were the most obvious misdiagnoses.

**Table 3:** The patient’s clinical presentation before and during the doctor’s visit of our department.

No.	Initial symptoms	Duration	Symptoms at first visit to a doctor	Duration	First Diagnosis	Misdiagnose	No. of visits	Duration
1	Increased frequency of stools, loose stools, minor left lower abdominal discomfort, Weight loss for 1 year	6 months	A mass appeared in the left lower abdomen with intermittent left lower abdominal pain	1 week	Descending colon cancer with retroperitoneal abscess	no	2	3 day
2	no	no	Fluctuating mass in the left lower abdomen	1 days	Abdominal abscess	yes	2	19 days
3	no	no	Pain in the right lower quadrant	1 week	Periappendicural abscess	yes	5	10 months
4	The left groin was sore	11 days	The left groin soreness worsened	1 days	muscular strain	yes	2	2 weeks

The first duration indicates that the patient did not pay attention and seek medical attention after the onset of symptoms, the second indicates the duration of symptoms before the patient sought medical attention; The third represents the time from the time the patient first visited the hospital to the time he arrived at our department. No. of visits indicates number of hospital visits of patients before coming to our department.

### 3.3 Laboratory examination

**Stable 2** summarizes the laboratory examination results of the four patients from our department. Among the four patients, the patients of case 1 and case 4 had elevated white blood cells, and all four patients presented with symptoms of procalcitonin elevation and anemia with decreased red blood cells and hemoglobin. Three of the patients had elevated tumor markers and in two patients, *Escherichia coli* was cultured. **Stable 3** summarized the results in the literature, in which elevated leukocytes were reported in 87.5% of the patients, anemia was reported in 87.5% (14/16), C-reactive protein (CRP) and ESR were elevated in 95.4% (21/22) and 63.6% of patients, respectively, and tumor markers CEA and CA199 were elevated in 53.8% and 57.1% of patients, respectively. The results of bacterial culture were reported in 19 patients, with 47.36% (9/19) reporting *Escherichia*, 26.3% (5/19) of *Streptococcus*, and 15.8% (3/19) reporting *Proteus vulgaris*.

### 3.4 Imaging interpretation of the abscesses

**Figure 1:**
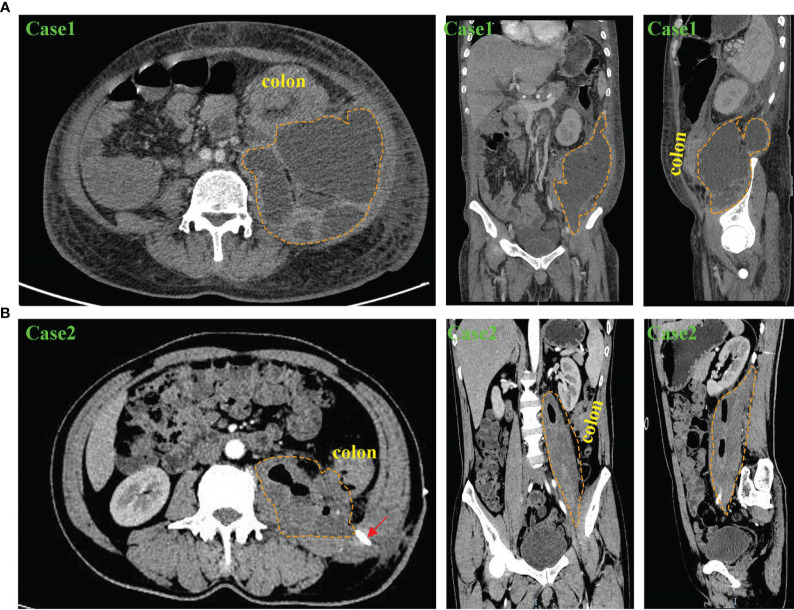
Abdominal and pelvic CT scan results of patients with retroperitoneal abscess in our department. **(A)** CT findings of Case 1 in transverse, coronal and sagittal planes. The results showed a huge right retroperitoneal and iliopsoas abscess with thickening of the descending colon wall. The yellow dashed circle shows the extent of the abscess. **(B)** CT findings of Case 2 in transverse, coronal and sagittal planes. The results showed a right retroperitoneal iliopsoas abscess and thickening of the descending colon wall. The yellow dashed circle shows the extent of the abscess, and the red arrow shows the drainage tube for percutaneous drainage.

**Figure 2:**
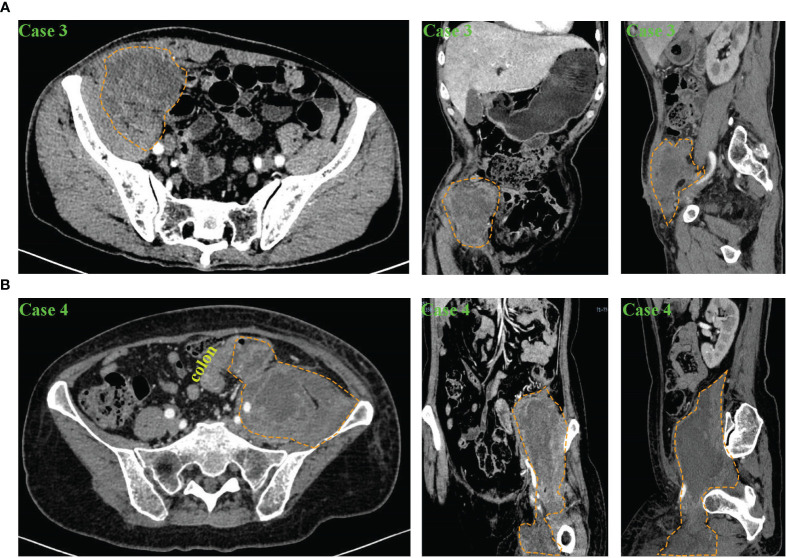
Abdominal and pelvic CT scan results of patients with retroperitoneal abscess in our department. **(A)** CT findings of Case 3 in transverse, coronal and sagittal planes. The results showed a left retroperitoneal abscess involving part of the abdominal wall and the iliacus muscle. The yellow dashed circle shows the extent of the abscess. **(B)** CT findings of Case 4 in transverse, coronal and sagittal planes. The results showed a right retroperitoneal abscess involving the iliopsoas muscle and the adductor muscle group. The yellow dashed circle shows the extent of the abscess.

**Figure 3:**
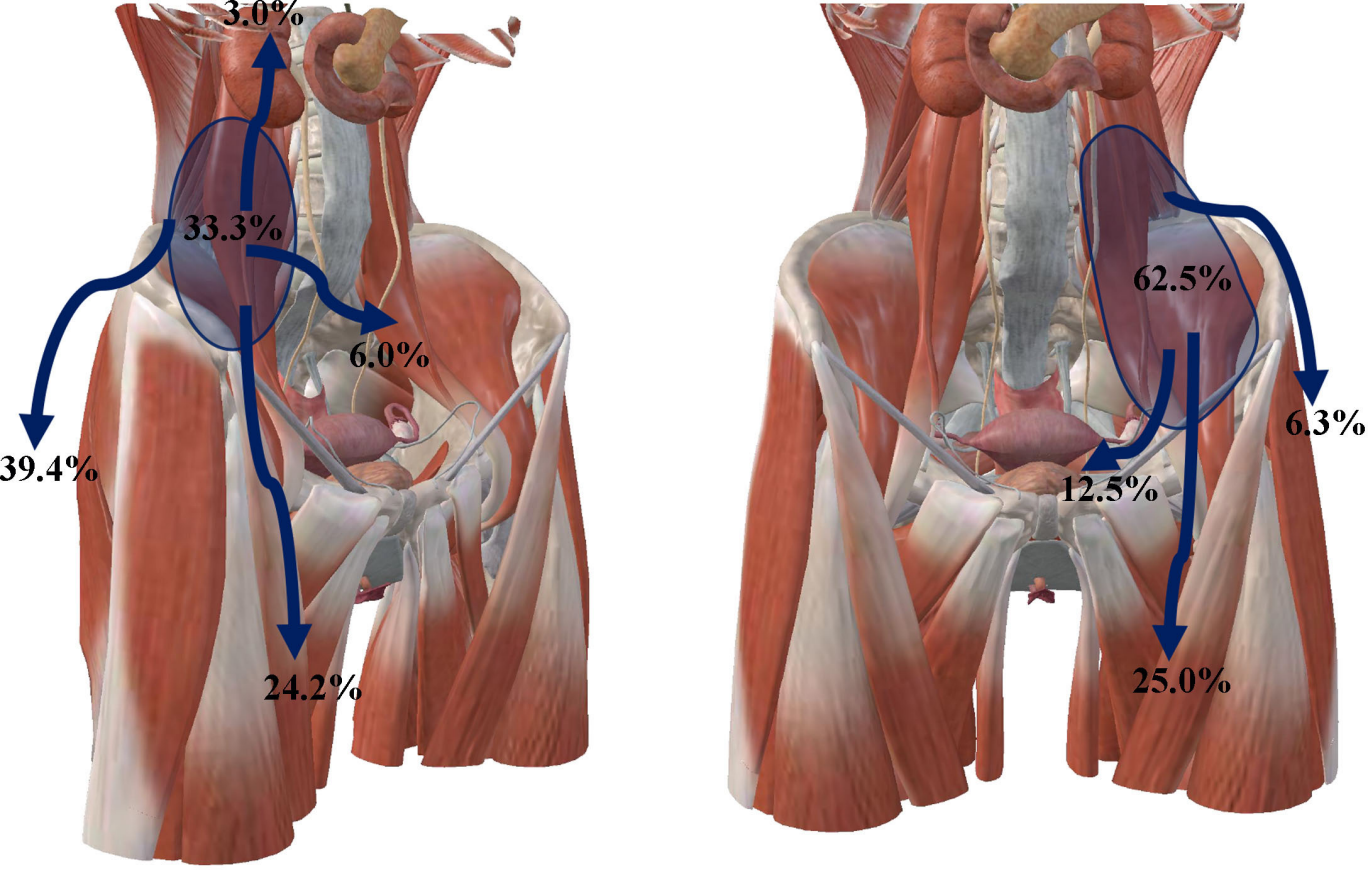
Proportion of the direction of abscess involvement in all cases in this article, the left side shows the direction of retroperitoneal abscess involvement caused by tumors in the ileocecum, appendix, and ascending colon, including the anterior pararenal space and perirenal space, local iliopsoas muscle, peritoneal penetration into the abdominal cavity, adductor muscle group, and subcutaneous tissue of the buttock and thigh after abscess passes through the weakness of the posterior abdominal wall. The right side shows the direction of retroperitoneal abscess involvement from descending colon cancer, including the region of the iliopsoas muscle, the pelvic retroperitoneum, the adductor muscle group, and subcutaneous tissue of the buttock and thigh after abscess passes through the weakness of the posterior abdominal wall.

**Figures 1**, **2** show the abscesses of our four patients and all patients were confirmed to have retroperitoneal abscess through CT. The abscess of the patients in case 1 extended up to the posterior pararenal space and down to the psoas over the suprapubic border. The left side was the lateral aspect of the lumbar cone at the origin of the psoas major muscle, the right side was the lateral peritoneum, and the anterior side was the ascending colon tumor (**Figure 1A**). The initial local hospital imaging findings of the patients of case 2 were essentially the same as those in case 1, and CT scans obtained after drainage in our hospital showed that the abscess was predominantly an iliopsoas abscess (**Figure 1B**). Case 3 was an ileocecal tumor that invaded the anterior peritoneum and abdominal wall, and the abscess mainly traveled along the iliac muscle and psoas muscle to the superior border of the femoral triangle (**Figure 2A**). The abscess of the patient in case 4 was the largest, traveling along the iliopsoas muscle to the adductor muscle group space (**Figure 2B**), and the abscess in all four patients contained the iliopsoas muscle (100%). After analyzing the 57 patients in the case reports, almost all patients had CT examination, X-ray, MRI, ultrasound and intestinal contrast examinations as the other major tests. **Stable 4** lists the CT findings of all patients, In forty-seven patients, the condition of the iliopsoas muscle was mentioned, 87.2% (41/47) of these patients had iliopsoas abscesses; and only 12.8% (6/47) of retroperitoneal abscesses did not have iliopsoas infections. These abscesses often contained gas; 35.1% (20/57) of patients had mild or severe skin injuries to the lower back, buttocks, and legs. In the CT report results, the intestinal condition was mentioned in 43 of the patients; there was no simultaneous detection of tumors during the process of finding an abscess on CT in 37.2% (16/43) of patients; and suspicious masses were found at the same time in 62.8% (27/43) of patients. Among them, 51 had partial or specific descriptions of abscesses, and 33.9% (18/53) mainly had local abscesses of the iliopsoas muscle. Among them, 26.4% (14/51) of patients had abscesses that mainly penetrated the weak points of the lower back and drained into the subcutaneous tissues of the waist and upper buttocks, and 92.9% (13/14) of these patients had tumors in the ileocecal region and ascending colon region. In 22.6% (12/53) of patients, the abscess of the iliopsoas muscle flowed through the inguinal ligament to the adductor group of the thigh or the subcutaneous tissue of the thigh, 33.3% (4/12) of these patient tumors were located in the descending colon and sigmoid colon, and 66.7% (8/12) were located in the ascending colon and ileocecal tumors. One (1.9%) had an abscess that drained to both the adductor muscle group and the retroperitoneal pelvis, and 2 (3.7%) had abscesses penetrating the peritoneum into the abdominal cavity, **Figure 3** shows the proportion of drainage in each direction for the retroperitoneal abscesses caused by the right and left colon cancer. Abscesses caused by left colon cancer appear to be more likely to form local abscesses, while abscesses caused by right colon cancer tend to drain to the subcutaneous tissue of the waist and buttocks. Based on our cases, the highest location of the abscess is at the originate point of the iliopsoas muscle and the lowest is from the adductor group to the knee.

### 3.5 Treatment and prognosis

**Figure 4:**
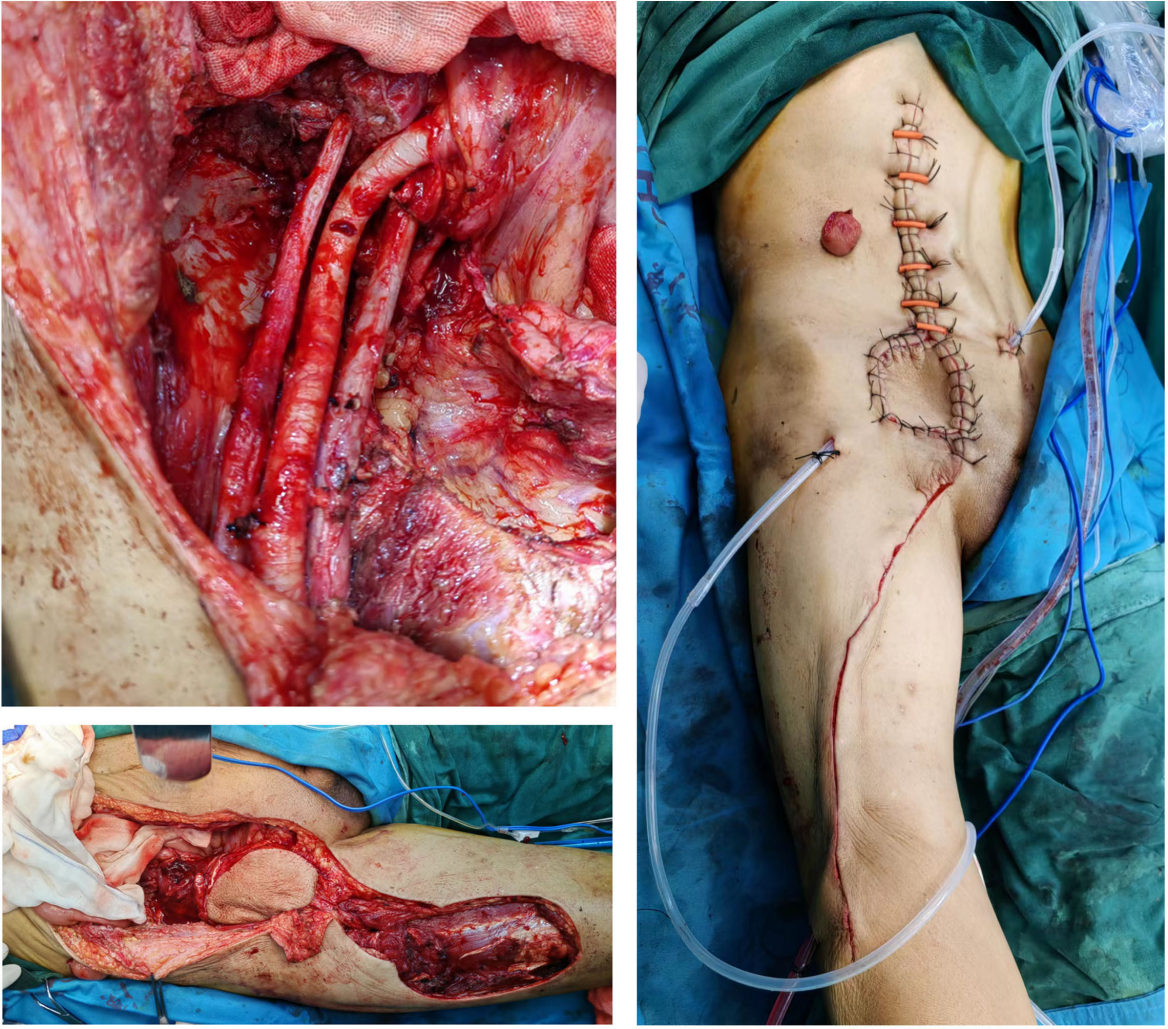
Intraoperative picture of Case 3, in which part of the iliopsoas muscle and part of the abdominal wall tissue were removed, and the deficient abdominal wall tissue was repaired by thigh flap transplantation.

As shown in **Table 4**, the patient in Case1 underwent ultrasound assisted drainage and anti-inflammatory therapy. After 8 days, an exploratory laparotomy was performed. The exploration found that intestinal resection and biopsy were extremely difficult and dangerous. A transverse colostomy was performed. After admission, the patient underwent endoscopy twice and laparotomy once, but the biopsy did not confirm a diagnosis. Finally, a clear diagnosis was made through CT guided puncture biopsy and genetic testing confirmed MSI-H. The patient received 5 cycles of CapeOX chemotherapy plus 7 cycles of Karelizumab immunotherapy and 1 time of radiotherapy starting from 1 month after surgery. The efficacy of the comprehensive treatment was evaluated as PR. Finally, the patient underwent laparoscopic radical descending colectomy plus transverse colon and transverse colon anastomosis. The postoperative pathology was ypT0N0M0. Immunotherapy was continued after surgery, and there was no recurrence after 9 months of follow-up. The treatment of the patient of Case 2 was basically the same as that of the patient of case 1. Before entering our department, the patient was diagnosed with an abscess and had puncture drainage in the local hospital, but the symptoms were not relieved. After being admitted to our department, we continued to drain and to provide medications to resist infection. The time for drainage and antiinflammatory therapy was long, and the pathological result was MSS-type bowel cancer. Currently, the patient is undergoing third-stage chemotherapy and targeted treatment, and the tumor has retreated. The patients of Cases 3 and 4 were operated on directly, with a large surgical wound and a long surgical time. The patient of Case 3 underwent multidisciplinary collaborative surgery, including colon tumor resection and small intestinal end ostomy, partial iliopsoas muscle resection, and abdominal wall musculocutaneous flap transfer (**Figure 4**). The patient developed slight lower limb numbness after surgery. The patient of Case 4 underwent palliative partial sigmoidectomy, sigmoidostomy, and incision and drainage of the iliac fossa abscess. The drainage time and treatment interval for cases 3 and 4 were short, and the patient in case 3 refused chemotherapy after surgery. After a follow-up of 3 months, there was no tumor recurrence. The patient of Case 4 received chemotherapy after surgery, and underwent ostomy after 1 year and 6 months. There was no recurrence after 1 year and 7 months of follow-up. In the literature, due to the difficulty in diagnosis and susceptibility to a misdiagnosis of this disease, there is no consistency in the treatment of all patients. The treatment is mainly focused on the treatment of abscesses and tumors. A total of 55.6% (25/45) of patients underwent ≥ 2 invasive procedures, including surgical debridement and surgery. If the abscess is treated first, the main method is percutaneous puncture drainage, and the treatment time is > 2 weeks. The prognosis was mention for thirty-four patients, and there were 13 deaths. Analysis of the causes of death showed that six patients died during hospitalization, including four patients who underwent direct surgery. Of these four patients, two underwent intestinal anastomosis, and two were poorly treated for abscesses. Seven other patients died after discharge due to cachexia, more details can be found in **Stable 5**.

**Table 4:** Treatment characteristics of 4 patients from our department.

No.	1	Interval time	Drainage tube indwelling time	2	Interval time	3	Interval time	4	prognosis
1	Ultrasound guided percutaneous puncture drainage	8d	2 months	laparotomy and transverse colostomy	1 month	Chemotherapy and/or immunotherapy	6 months	Laparoscopic radical resection of left colon cancer, transverse colon and transverse colon anastomosis, transverse colon and rectal anastomosis	No recurrence for 9 months
2	Ultrasound guided percutaneous puncture drainage	1 month	1 months	transverse colostomy	1 month	Chemotherapy and targeted therapy	/	/	In treatment
3	Anti-inflammatory therapy	3 d	2 weeks	Palliative tumor resection plus retroperitoneal abscess drainage	45 d	Chemotherapy	18 months	Closure of sigmoidostomy	No recurrence for1.9 year
4	Anti-inflammatory therapy	3 d	1 week	Palliative right hemicolectomy and terminal ileal resection,Terminal ileostomy,Musculo-flap transfer peritoneal repair,Partial iliopsoas resection	/	/	/	/	No recurrence for 3 months

## 4 Discussion

Retroperitoneal abscess is a rare complication in colorectal cancer patients, and the specific incidence is unclear. Our hospital probability of occurrence is approximately 0.39%, which is consistent with the 0.3% reported in other studies (4, 7). Due to the low incidence, to date, reports of colorectal cancer associated with retroperitoneal abscess have only been reported in case report literature, and to our knowledge, this is the first comprehensive analysis of the largest dataset of colorectal cancer associated with retroperitoneal abscess. Through the analysis of 61 patients, we found that colon cancer associated with retroperitoneal abscess often occurred in elderly patients aged > 60 years, with slightly more men than women; and the occurrence of the disease did not vary regionally; and the disease was reported worldwide. Right colon cancer, particularly malignancy tumors in the ileocecal region, is the most common location for this clinical sign. However, considering that the incidence of left colon cancer is generally lower than that of right colon cancer (54), the overall incidence of left colon cancer associated with retroperitoneal abscess is not lower than that of right colon cancer. Pathologically, almost all these patients have advanced colorectal adenocarcinoma.

Clinically, patients with colon cancer are admitted to hospitals frequently with hematochezia, abdominal pain, and the changes in defecation habits (55), whereas patients with retroperitoneal abscesses often have masked and atypical symptoms, and gastrointestinal symptoms are often absent. Because retroperitoneal symptoms are not as pronounced as those of intraperitoneal perforation, in this study, we found that some patients had insidious symptoms of fever and weight loss in the early stage, and these patients often do not choose to seek medical attention because the symptoms are atypical. Most patients were admitted to hospitals with corresponding discomfort due to retroperitoneal abscess or iliopsoas abscess, most notably pain and swelling in the thighs, abdomen, and lower back. The misdiagnosis rate of this disease is high. Of the 4 patients received by our department, three patients were misdiagnosed at their initial visit at local hospital, while the misdiagnosis rate of the cases extracted from the literature was 43.9%. The reasons for the misdiagnose are manifold, with two directions being the core causes. First, due to the lack of digestive system symptoms, patients often seek medical advice due to pain, which leads to errors in the initial visit and a lack of a systematic examination. Second, there are various causes of retroperitoneal abscess or iliopsoas muscle abscess, such as tuberculosis of the spine, Crohn’s disease, appendicitis, diverticulitis, urinary tract infection, and cancer (56, 57). Diseases of the digestive system are not the core cause, and some patients often receive emergency admission (58, 59). Therefore, in these patients, the main focus is on treating their abscess and subcutaneous necrotic diseases, while ignoring the primary disease after admission. In addition, early diagnosis of retroperitoneal abscess is sometimes difficult because the retroperitoneal space is relatively invisible to the examiner. When an abscess forms within the abdominal cavity, the patient can often quickly feel infection due to the sensitivity of the peritoneum, and symptoms of peritoneal irritation, such as tenderness and muscle tension, can be exhibited. When the abscess forms only in the retroperitoneal location, the symptoms are mild and atypical. Therefore, in clinical practice, systematic thinking and the exclusion of diagnoses should be performed for patients with retroperitoneal abscess, and a discussion within an MDT is particularly important. Misdiagnosis not only delays the treatment of patients, but also affects their prognosis due to tumor progression.

The most obvious laboratory indicator in patients with colorectal cancer and retroperitoneal abscess is an increase in inflammation related indicators, including white blood cells, CRP, and procalcitonin. Anemia is another important characteristic, that may be related to tumor necrosis and bleeding, repeated inflammatory stimulation, and body consumption. In this article, we found that colorectal and retroperitoneal abscesses did not occur suddenly. Hidden symptoms such as fever and weight loss often persist for a long time, and substances such as fecal fluid are often not detected in the abscess drainage fluid. These findings suggest that retroperitoneal abscess caused by colorectal cancer may be caused by the translocation of intestinal flora to the retroperitoneum through small perforations caused by tumor necrosis and damaged intestinal barriers. The bacterial culture of pus confirmed that the bacterial population was mainly Escherichia coli from the intestine. It is worth noting that more than 50% of patients have elevated tumor markers for CEA and CA199, indicating that these patients are mostly diagnosed with mid- to late-stage tumors. When the etiology of retroperitoneal abscess is difficult to diagnose, elevated tumor markers can be an important reference indicator for the origin of colorectal cancer.

Compared to clinical symptoms and laboratory examinations, imaging methods such as CT, MRI, ultrasound, and X-ray imaging are the core examination methods for colorectal cancer associated with retroperitoneal abscess. In this paper, based on the results of the CT examinations, we found that patients with retroperitoneal abscess due to colorectal cancer often have abscesses with a large range of sizes, which can compress tissue., resulting in intestinal masses that are not easy to find, and this is also an important cause of misdiagnose in patients. Through the CT analysis of large datasets and the imaging description of the posterior peritoneum by A. Coffin et al. (3), we statistically found that the main distribution areas of retroperitoneal abscess caused by colon cancer were the pararenal posterior space and the posterior iliopsoas muscle retroperitoneal chamber, and the pararenal anterior space and the perirenal space were affected in only one patient. Retroperitoneal abscesses due to colon cancer are mainly presented iliopsoas muscles (100% of our case and 87.2% of the cases in the literature). The psoas major muscle extends from T12 to the trochanter, which may be affected by gravity, and most of the abscess flows down the psoas major muscle, resulting in a local abscess of the iliopsoas muscle, the thigh adductor group and subcutaneous tissue around the end of the psoas muscle. In addition, drainage of the abscess through the anatomical weakness of the lumbar hernia to the upper buttocks and subcutaneous tissue of the lumbar spine is another important method, and it is worth noting that right colon cancer is more likely to cause subcutaneous abscesses due to anatomical factors. There is a certain difference between peritoneal abscesses formed by colorectal cancer and other peritoneal abscesses, abscesses formed by colon cancer often take a certain amount of time to develop, while retroperitoneal abscesses caused by diverticula are often suddenly, with their retroperitoneal diffusion being wider (60).

Currently, the treatment methods for retroperitoneal abscess caused by colorectal cancer vary, and different treatment methods will lead to completely different treatment outcomes for such patients. Here, case reports and our cases are combined for illustration, and the best treatment methods for such patients are discussed. The first treatment method, simple incision and drainage plus anti-inflammatory treatment, can improve the symptoms of the abscess in a short time, but because the primary disease is not treated, the patient may experience repeated symptoms, enterocutaneous fistula formation, skin damage, multiple hospitalizations, and surgery. However, drainage can create opportunities for subsequent surgery. Through drainage, many abscess symptoms disappeared. At this time, a clear diagnosis can be made and surgical treatment can be performed. The second way is for the tumor to invade the posterior peritoneum, with or without microabscesses. If only medical treatment of the tumor is carried out first, it can lead to abscess formation, abscess spread, increased infection, limited limb activity, and necrotizing fasciitis. The third method is direct colon resection and intraopertive (41) debridement and drainage. However, due to the presence of an abscess, incision of the posterior peritoneum may cause the abscess to spread to the abdominal cavity, tumor resection may not be complete, and the patient may not be able to tolerate surgery. In our cases, two patients underwent direct surgical treatment. Although one patient survived after 2 years of follow-up, the surgical wound was large, and the surgery was difficult, and may required multidisciplinary cooperation. The fourth method involves comprehensive treatment such as anti-inflammatory and abscess drainage, proximal enterostomy, radiotherapy and chemotherapy, plus radical surgery and ostomy, as well as postoperative comprehensive treatment. This method takes a long time, but it can achieve radical resection. Several scholars have reported this scheme with obvious results (37, 41, 48). In our case series, this method was adopted for tow patients, and the current treatment effect is good. Although the drainage time of patients undergoing direct surgery is significantly shortened, and some patients can achieve long-term survival, according to literature reports, direct extended surgery is also an important cause of death. The specific reason may be that patients cannot tolerate large-scale surgery, and intestinal anastomosis is not encouraged during surgery, which may lead not only to anastomotic leakage, but also to patient death. It is worth noting that if comprehensive treatment is performed, it is necessary to obtain specimens for pathological examination and genetic testing before surgery. In the case of abscess, it is sometimes difficult to obtain tumor tissue through colonoscopy, and colonoscopy is performed from the stoma end after stoma. CT and ultrasound guided localization can be used as better alternatives. The author does not recommend performing laparotomy and endoscopic biopsy, which not only easily leads to pus contamination of the abdominal cavity, but also leads to tumor dissemination in the abdominal cavity.

In summary, colon cancer combined with retroperitoneal abscess often has hidden symptoms of fever and weight loss. The clinical manifestations are mainly pain in the thigh, abdomen, and back caused by an iliopsoas muscle abscess. When patients have these symptoms, they need to undergo systematic evaluation, tumor markers testings, CT, and other examinations to rule out the possibility of colon cancer. Clear and timely diagnosis is the core of treatment for such patients. In terms of treatment, anti-inflammatory agents and abscess drainage are the primary treatment items. Proximal enterostomy can not only alleviate inflammation and abscesses, but also create conditions for comprehensive treatment. After comprehensive treatment, selecting the opportunity to perform radical tumor surgery and stoma reduction surgery may be the best treatment plan.

## Data availability statement

The original contributions presented in the study are included in the article/**Supplementary Material**. Further inquiries can be directed to the corresponding authors.

## Ethics statement

This study has been approved by Ethical Approval for Clinical Research Projects under Medical Ethics Committee, Zhongnan Hospital of Wuhan University (2021061). The studies were conducted in accordance with the local legislation and institutional requirements. Written informed consent for participation was not required from the participants or the participants’ legal guardians/next of kin in accordance with the national legislation and institutional requirements. Written informed consent was obtained from the individual(s) for the publication of any potentially identifiable images or data included in this article.

## Author contributions

DL and HY conceived and designed the study. JZ and SW performed the experiments and the statistical analysis. JZ, CL, QQ and ZD participated in the discussion and interpretation of the data. DL and JZ wrote the manuscript. DL and JZ read the literature. CL, QQ and ZD performed surgical treatment. DL and HY confirmed the authenticity of all the raw data. All authors contributed to the article and approved the submitted version.
